# Heterogeneity Among Poor Ovarian Responders According to Bologna Criteria Results in Diverging Cumulative Live Birth Rates

**DOI:** 10.3389/fendo.2020.00208

**Published:** 2020-04-16

**Authors:** Alessia Romito, Erlisa Bardhi, Joaquin Errazuriz, Christophe Blockeel, Samuel Santos-Ribeiro, Michel De Vos, Annalisa Racca, Shari Mackens, Annelore Van Der Kelen, Pierluigi Benedetti Panici, Alberto Vaiarelli, Herman Tournaye, Panagiotis Drakopoulos

**Affiliations:** ^1^Centre for Reproductive Medicine, Universitair Ziekenhuis Brussel, Brussels, Belgium; ^2^Department of Maternal and Child Health and Urological Sciences, Sapienza University of Rome, Rome, Italy; ^3^Departamento de Ginecologia y Obstetricia Clinica Alemana, Facultad de Medicina, Universidad del Desarrollo, Santiago, Chile; ^4^Department of Obstetrics and Gynaecology, University of Zagreb, Zagreb, Croatia; ^5^Instituto Valenciano de Infertilidade, IVI-RMA Lisboa, Lisbon, Portugal; ^6^Clinica Valle Giulia, G.E.N.E.R.A. Centre for Reproductive Medicine, Rome, Italy

**Keywords:** Bologna criteria, poor ovarian response, poor responders, cumulative live birth rate, ICSI

## Abstract

**Research Question:** Does reproductive outcome differ among the various subgroups of poor ovarian responders according to the Bologna criteria?

**Design:** This was a retrospective, cohort study including poor ovarian responders according to Bologna criteria, undergoing an ICSI cycle from January 2011 until December 2017. Patients were divided into four groups: (1) age ≥ 40 years and abnormal ovarian response test, (2) age ≥ 40 years, abnormal ovarian reserve test and one previous poor response to stimulation, (3) age ≥ 40 years and one previous poor response, (4) abnormal ovarian reserve test and one previous poor response.

**Result(s):** Overall, 846 cycles in 706 Bologna poor ovarian responders were included: 310 cycles in group 1, 169 in group 2, 52 in group 3, and 315 in group 4. There were significant differences in age, antral follicle count, antimüllerian hormone, cycle cancellation rates, and number of retrieved oocytes between the four groups. Live birth and cumulative live birth rate differed significantly between groups and were highest in Group 4 [Live birth rate: 7.4% (1) vs. 4.1% (2) vs. 5.8% (3) vs. 13.4% (4), *p* = 0.001 and Cumulative live birth rate: 8.3% (1) vs. 4.1 % (2) vs. 9.6% (3) vs. 16.8% (4) *p* < 0.001]. The multivariate GEE analysis revealed that the number of MIIs and the Bologna criteria pattern were the variables which were significantly associated with cumulative live birth rate.

**Conclusion(s):** Poor ovarian responders represent a heterogeneous population. The young subpopulation has a better clinical prognosis in terms of fresh and cumulative live birth rate.

## Introduction

The occurrence of poor ovarian response amongst patients undergoing ovarian stimulation for *in vitro* fertilization (IVF) ranges from 9 to 24%, meaning that almost one in four patients harbors a poor reproductive prognosis (1–4). This category of patients commonly referred to as poor responders (PORs), remains a challenge for the fertility expert, as studies up to now have failed to identify a therapeutic approach that can modify or improve their fertility outcomes (5–7). The lack of conclusive evidence is strongly related to the vast heterogeneity of criteria that until recently were used to define PORs, which made it impossible to compare studies among them and develop valid, evidence-based management strategies (8). Indeed, until a few years ago, a myriad of vague and mostly arbitrary definitions of POR were used (9). The first attempt to overcome this hurdle, by accurately defining PORs in a standardized way, was carried out in 2011 by the European Society for Human Reproduction and Embryology (ESHRE) with the introduction of the Bologna Criteria (BC) (10). In the definition of POR by the BC, at least two of the following features must be present: advanced maternal age (≥40 years), a previous poor ovarian response with ≤ 3 oocytes retrieved after conventional stimulation and/or an abnormal ovarian reserve test (ORT) [i.e., antral follicle count (AFC) <7 or antimüllerian hormone (AMH) <1.1 ng/ml]. In the absence of advanced maternal age or abnormal ORT, a patient can be defined as POR after two episodes of poor ovarian response following maximal stimulation. The BC have thus inevitably reduced the heterogeneity previously present in the definition of POR and certainly represent a key step in framing the characteristics that better affiliate with this difficult setting of patients. However, there is evidence that even within the BC population, a significant degree of heterogeneity still persists (11). Indeed, several patterns or subgroups of PORs can be distinguished within the BC based on possible combinations of risk factors, ORT results, and IVF attempts (11–13). These subpopulations clearly encompass diverse baseline characteristics and the prognostic potential of these naturally emerging subgroups, as well as their reproductive outcomes, remain unclear and urge further investigation. These discrepancies within BC patients may explain why the few randomized controlled trials (RCTs) conducted in BC POR did not show a benefit of any treatment modality in terms of live birth rates (LBR). Furthermore, although LBR following a fresh IVF cycle is a key outcome measure for treatment success, cumulative LBR after the utilization of all fresh and frozen–thawed embryos derived from one stimulation cycle, has emerged as a more clinically meaningful outcome. In this context, the aim of our study was to evaluate cumulative LBR in different patterns of BC PORs.

## Materials and Methods

### Study Design

This was a retrospective, single-center cohort study including PORs according to BC and undergoing an intracytoplasmic sperm injection (ICSI) cycle, at the Centre for Reproductive Medicine, Universitair Ziekenhuis Brussel, Belgium, from January 2011 to March 2017. This study was approved by the Ethics Committee of Brussels University Hospital (approval B.U.N. 143201938863).

### Eligibility Criteria

Clinical data regarding all ICSI cycles using a fixed antagonist protocol and gonadotrophin dose of at least 300 IU per day were collected. Patients were included if they fulfilled at least two of the following criteria: advanced maternal age (≥40 years), abnormal ORT (AMH <1.1 ng/ml or AFC <7) or a previous poor ovarian response (≤ 3 oocytes with a conventional stimulation protocol). Additional inclusion criteria were: age between 18 and 43 years, body mass index (BMI) of 17–35 kg/m^2^, presence of both ovaries and absence of any untreated endocrine abnormality. Women were excluded if they underwent preimplantation genetic testing (PGT).

Women who fulfilled the aforementioned criteria but, for unknown reasons, still had frozen embryos remaining or who had transferred the remaining embryos to another IVF unit, while not delivering a live born following their stimulated ICSI cycle, were excluded from the analysis in order to minimize the risk of misclassification bias. All patients had a follow up of at least 2 years.

### Bologna Criteria Patterns

The population of patients that fulfilled these eligibility criteria was divided into four different patterns: (1) age ≥40 years and abnormal ORT, (2) age ≥40 years, abnormal ORT and one previous poor response to stimulation, (3) age ≥40 years and one previous poor response, (4) abnormal ORT and one previous poor response to stimulation (11).

### AMH Analysis

AMH testing was performed between one and 3 months prior to the start of the ART cycle. Until the 24th of April 2012, our center used the AMH Immunotech (IOT) kit (Beckman Coulter Inc., Marseilles, France). Between the 25th of April 2012 and 3rd of July 2013, the Gen II kit was used (Beckman Coulter, Inc., Chaska, Minnesota, USA); between the 4th of July 2013 and 17th of September 2014 the modified Gen II test kit was used and since the 18th of September 2014 the Elecsys platform (Roche Diagnostics International AG, Rotkreuz, Switzerland) has been used. To homogenize AMH levels across these time intervals we used published conversion formulas (14, 15).

### Treatment Protocol

From day 2 or 3 of the menstrual cycle all patients received fixed daily doses of ≥300 IU of highly purified human menopausal gonadotropin (hp-hMG) or recombinant follicle stimulating hormone (FSH) until the day prior to human chorionic gonadotrophin (hCG) administration.

Pituitary suppression was achieved with daily administration of a gonadotropin releasing hormone (GnRH) antagonist starting from day 6 of stimulation onwards. Women did not use oral contraceptives or estrogen priming prior to ovarian stimulation. A blood sample to measure estradiol (E2), progesterone (P), FSH, and luteinizing hormone (LH) levels and an ultrasound was performed on Day 2 or 3 of the menstrual cycle and from day 7 or 8 of the cycle onwards. Triggering of final oocyte maturation was achieved using 5,000 IU highly purified urinary or recombinant hCG, when at least two follicles reached 17 mm in mean diameter. In case of mono-follicular development, patients were given the option to proceed to oocyte retrieval nonetheless. All patients of our cohort with monofollicular development proceeded with oocyte pick-up. Cycle was canceled if there was no follicles development after 10 days of stimulation. Cumulus-oocyte complexes (COCs) were collected by transvaginal aspiration 36 h after hCG administration, followed by insemination via ICSI (16). Luteal phase support consisted of vaginal micronized progesterone (200 mg, three times a day), initiated the day after the oocyte retrieval and continued until at least 7 weeks, in case of a positive pregnancy test.

### Embryo Transfer

On day 3 or 5 after oocyte retrieval an ultrasound-guided fresh embryo transfer (ET) was performed. The day of the transfer was chosen in accordance with our internal policy; specifically, when at least 4 embryos of top quality (at least 7 cells with maximum 10% fragmentation) or good quality (at least 6 cells with maximum 20% fragmentation) were present on Day 3, embryo culture was extended to Day 5, followed by fresh ET on Day 5. Otherwise, ET took place on Day 3. The maximum number of embryos transferred in the fresh cycle was limited to three.

### Cryopreservation

Vitrification of supernumerary good quality embryos was performed on Day 3 or Day 5 using closed blastocyst vitrification high security straws combined with dimethylsulphoxide and ethylene glycol bis (succinimidyl succinate) as the cryoprotectants (17). Good-quality Day 3 embryos were defined as embryos that reached the 6-cell stage with <20% fragmentation. Good-quality Day 5 embryos were defined as having trophectoderm and inner cell mass quality scores of at least AB, BA, or BB (18).

### Frozen–Thawed Embryo Transfer

Frozen ET, following warming of vitrified embryos, was performed either in a natural cycle, with or without hCG triggering, or in an artificial cycle, as previously described elsewhere (19). The decision regarding the type of preparation for the frozen ET cycle was made by the physician, based on the menstrual cycle pattern of the patient. The number of embryos transferred in the frozen-thawed cycles was one or two in compliance with Belgian regulatory guidelines and according to the patients' individual preference.

### Primary Outcome

The primary outcome was cumulative LBR defined as the number of deliveries with at least one live birth (>22 weeks of gestation) resulting from one initiated or aspirated ART cycle, including all cycles in which fresh and/or frozen embryos were transferred, until one delivery with a live birth occurred or until all embryos were used, whichever occurred first (20).

### Secondary Outcomes

The secondary outcomes included biochemical pregnancy rate (BPR, a pregnancy diagnosed only by the detection of hCG in serum or urine and that does not develop into a clinical pregnancy), clinical pregnancy rate (CPR, a pregnancy diagnosed by ultrasonographic visualization of one or more gestational sacs), ongoing pregnancy rate (OPR, diagnosed by ultrasonographic visualization of an intrauterine sac with embryonic pole demonstrating cardiac activity at 10 weeks of gestation), and LBR following fresh ET (delivery of a live born after 22 weeks of gestation, following the fresh IVF/ICSI cycle only) (20).

### Statistical Analysis

Outcomes were analyzed by BC pattern. In order to ascertain patients' baseline characteristics and important aspects of the treatment, continuous data are presented as mean ± standard deviation (SD) and median-interquartile range (IQR), while categorical data are described by number of cases, including numerator and denominator, and percentages. Categorical data and continuous data that did not show a normal distribution were analyzed by Pearson's χ^2^-test/Fisher exact test or Kruskal–Wallis test as appropriate.

Furthermore, in order to assess the association between cumulative LBR and BC patterns after adjustment for relevant confounders, multivariable regression models with estimation by generalized estimating equations (GEE) were used in order to account for inclusion of multiple cycles of a patient. The potential predictors considered for the analysis were BMI, number of metaphase II (MIIs) oocytes and day/number of embryos transferred in the fresh cycle.

All statistical tests used a two-tailed α of 0.05. All analyses were performed using STATA 13.0 (StataCorp. Stata Statistical Software: Release 13. College Station, TX, USA).

## Results

### Baseline Characteristics

Overall, data from 846 cycles in 706 Bologna PORs were included in the analysis and divided into four different patterns: 310 cycles in pattern 1, 169 in pattern 2, 52 in pattern 3, and 315 in pattern 4. Patients' baseline characteristics among the four groups were similar regarding BMI and basal FSH. There were significant differences in female age (41.3 ± 1.2 vs. 41.4 ± 1.1 vs. 41.3 ± 1.0 vs. 35.3 ± 3.5, respectively, for the four patterns, *P* < 0.001), AFC (4.6 ± 2.7 vs. 4.4 ± 2.6 vs. 8.8 ± 4.2 vs. 4.6 ± 2.7, respectively, *P* < 0.001), and AMH (0.57 ± 0.29 vs. 0.54 ± 0.28 vs. 1.7 ± 0.6 vs. 0.5 ± 0.3, respectively, *P* < 0.001), among the four patterns. The baseline characteristics of patients are summarized in (Table 1).

**Table 1:** Baseline characteristics of patients.

	**Pattern 1** **Age ≥ 40 y** **AMH < 1.1** **(*n* = 310)**	**Pattern 2** **Age ≥ 40 y** **AMH < 1.1** **PS ≤ 3 COCs** **(*n* = 169)**	**Pattern 3** **Age ≥ 40 y** **PS ≤ 3 COCs** **(*n* = 52)**	**Pattern 4** **Age < 40 y** **AMH < 1.1** **PS ≤ 3 COCs** **(*n* = 315)**	***P*-value**
Age (years)	41 (40–42) 41.3 ± 1.2	41 (41–42) 41.4 ± 1.1	41 (40–42) 41.3 ± 1.0	36 (33–38) 35.3 ± 3.5	<0.001^a^
BMI (kg/m^2^)	24 (22–28) 25.1 ± 4.4	24 (22–28) 24.9 ± 4.3	24 (21–27) 24.2 ± 4.6	24 (22–28) 25.2 ± 4.7	0.483^a^
Basal FSH	10.1 (8.0–12.8) 10.8 ± 4.0	10.3 (7.7–13.3) 10.8 ± 4.6	9.6 (7.6–11.4) 9.6 ± 3.2	9.8 (7.4–12.4) 10.5 ± 4.4	0.389^a^
AMH (ng/ml)	0.55 (0.30–0.80) 0.57 ± 0.29	0.47 (0.29–0.78) 0.54 ± 0.28	1.5 (1.3–1.9) 1.7 ± 0.6	0.4 (0.3–0.7) 0.5 ± 0.3	<0.001^a^
AFC	4 (2–6) 4.6 ± 2.7	4 (2–6) 4.4 ± 2.6	8 (5–12) 8.8 ± 4.2	4 (3–6) 4.6 ± 2.7	<0.001^a^
PS attempts (*n*)	1 (0–2)	2 (1–4)	2 (1–4)	2 (1–3)	<0.001^a^

*PS, previous stimulation; COC, cumulus-oocyte complex; BMI, body mass index; FSH, follicle stimulating hormone; AMH, antimüllerian hormone; AFC, antral follicle count*.

a *Kruskal–Wallis test. Values are mean (SD) and median (IQR)*.

### Ovarian Response and Characteristics of Embryo Development

Among the different subgroups, the number of COCs retrieved was significantly different (3.5 ± 2.3 vs. 2.9 ± 1.8 vs. 3.8 ± 1.6 vs. 2.7 ± 1.7 for pattern 1, 2, 3, 4, respectively, *P* < 0.001). Similarly, Implantation rates were significantly different: 54 (17%) vs. 26 (11%) vs. 7 (8%) vs. 50 (22%) for pattern 1, 2, 3, 4, respectively, *P* = 0.001. Cycle cancellation rates differed also significantly between groups (25.3% vs. 21.9% vs. 17.3 vs. 31.4%, *P* = 0.041). The number of embryos transferred was higher in pattern 4 compared to the other patterns (1.4 ± 2.1 vs. 1.3 ± 0.9 vs. 1.6 ± 1.0 vs. 2.2 ± 1.5, *P* < 0.001). Most ETs (94%) took place on Day 3. These results are presented in (Table 2).

**Table 2:** Cycle characteristics of controlled ovarian stimulation.

	**Pattern 1** **Age ≥ 40 y** **AMH < 1.1** **(*n* = 310)**	**Pattern 2** **Age ≥ 40 y** **AMH < 1.1** **PS ≤ 3 COCs** **(*n* = 169)**	**Pattern 3** **Age ≥ 40 y** **PS ≤ 3 COCs** **(*n* = 52)**	**Pattern 4** **Age < 40 y** **AMH < 1.1** **PS ≤ 3 COCs** **(*n* = 315)**	***P*-value**
COCs	3 (2–5) 3.5 ± 2.3	3 (2–4) 2.9 ± 1.8	4 (2–5) 3.8 ± 1.6	3 (1–3) 2.7 ± 1.7	<0.001^a^
MII	3 (1–4) 2.9 ± 2.1	2 (1–4) 2.4 ± 1.6	3 (2–4) 3.1 ± 1.6	2 (1–3) 2.2 ± 1.5	<0.001^a^
Cycle with ET, *n* (%)	230 (74)	131 (76)	43 (83)	200 (64)	0.001^b^
Number of embryos transferred	1 (0–2) 1.4 ± 2.1	1 (1–2) 1.3 ± 0.9	2 (1–2) 1.6 ± 1.0	2 (1–3) 2.2 ± 1.5	<0.001^a^
Day of transfer, *n* (%)					
Day 3	219 (95)	126 (96)	43 (100)	178 (89)	0.005^c^
Day 5	11 (5)	5 (4)	0 (0)	22 (11)	
Cycle cancelation, *n* (%)	79 (25.3%)	37 (21.9%)	9 (17.3%)	99 (31.4%)	0.041^b^

*PS, previous stimulation; AMH, antimüllerian hormone; COC, cumulus oophorus complex; MII, metaphase II oocytes; ET, embryo transfer*.

a *Kruskal–Wallis test. Values are mean (SD) and median (IQR)*.

b *Pearson χ^2^-test. Values are number (percentage)*.

c *Fisher exact test. Values are number (percentage)*.

### Reproductive Outcomes

**Figure 1:**
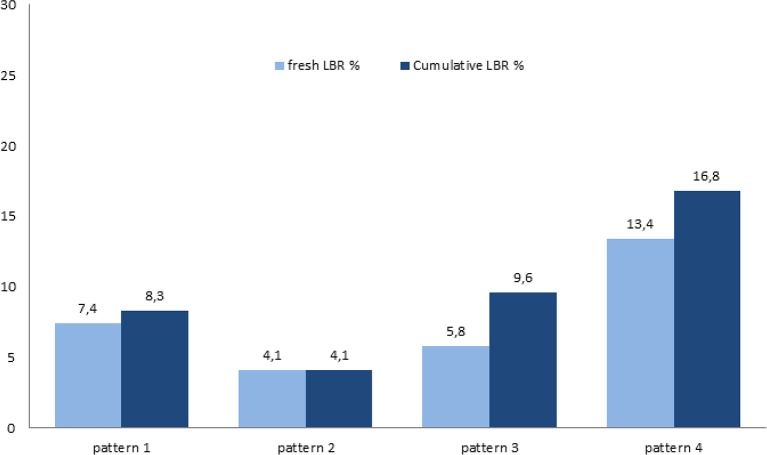
Fresh and cumulative live birth rates in different POR patterns.

Reproductive outcomes are displayed in (Table 3). There was no statistically relevant difference in terms of BPR and CPR among different patterns. However, OPR (7.4% vs. 4.1% vs. 5.8% vs. 13.7%, respectively, for the four patterns, with *P* = 0.002), LBR (7.4% vs. 4.1% vs. 5.8% vs. 13.4%, respectively, with *P* = 0.001), and cumulative LBR (8.3% vs. 4.1% vs.9.6% vs. 16.8%, respectively with *P* < 0.001) significantly differed among the four patterns (Figure 1). In particular, the *P*-values for the unadjusted pairwise comparisons for cumulative LBR between patterns were as follow: pattern 1 vs. 2 (*P* = 0.09), pattern 3 vs. 1 (*P* = 0.8), pattern 4 vs. 1 (*P* = 0.002), pattern 3 vs. 2 (*P* = 0.13), pattern 4 vs. 2 (*P* < 0.001), pattern 4 vs. 3 (*P* = 0.19).

**Table 3:** Reproductive outcomes.

	**Pattern 1** **Age ≥ 40 y** **AMH < 1.1** **(*n* = 310)**	**Pattern 2** **Age ≥ 40 y** **AMH < 1.1** **PS ≤ 3 COCs** **(*n* = 169)**	**Pattern 3** **Age ≥ 40 y** **PS ≤ 3 COCs** **(*n* = 52)**	**Pattern 4** **Age < 40 y** **AMH < 1.1** **PS ≤ 3 COCs** **(*n* = 315)**	***P*-value**
Biochemical pregnancy rate, *n* (%)	60 (19.2)	31 (18.3)	9 (17.3)	58 (18.4)	0.985^b^
Clinical pregnancy rate, *n* (%)	54 (17.3)	26 (15.4)	7 (13.5)	50 (15.9)	0.879^b^
Ongoing pregnancy rate, *n* (%)	23 (7.4)	7 (4.1)	3 (5.8)	43 (13.7)	0.002^b^
LBR, *n* (%)	23 (7.4)	7 (4.1)	3 (5.8)	42 (13.4)	0.001^c^
Cumulative LBR, *n* (%)	26 (8.3)	7 (4.1)	5 (9.6)	53 (16.8)	<0.001^b^

*PS, previous stimulation; AMH, antimüllerian hormone; COC, cumulus-oocyte complex; LBR live birth rate (following the fresh embryo transfer); cumulative LBR (definition – to clarify the table when seen isolated from the text)*.

a *Pearson χ^2^-test. Values are number (percentage)*.

b *Fisher exact test. Values are number (percentage)*.

Categorization of patients in subgroups based on AFC (rather than AMH) resulted in similar findings (Supplementary Table 1).

Furthermore, in order to avoid selection bias (patients repeating a second cycle may have had a better prognosis), an extra analysis was performed: main reproductive outcomes were analyzed per patient (one cycle per patient). Results remained approximately the same (Supplementary Table 2).

### GEE Analysis for Cumulative LBR

The multivariate GEE analysis revealed that the number of MIIs (coefficient 0.03, *P* = 0.006) and the BC pattern [coefficients for pattern 1: (–), pattern 2: −0.04, pattern 3: 0.01, pattern 4: 0.14, *P* < 0.001] were the only variables which were significantly associated with cumulative LBR. *Post-hoc* pairwise comparisons revealed that pattern 4 was significantly associated with higher cumulative LBR compared to all the other patterns after adjustment for confounders: pattern 4 vs. 1 (*P* < 0.001), pattern 4 vs. 2 (*P* < 0.001), pattern 4 vs. 3 (*P* = 0.046). These findings are presented in (Table 4).

**Table 4:** GEE regression analysis for cumulative LBR.

**Cumulative LBR**	**Coefficient**	**Standard error**	***P*-value**
BMI	0.002	0.003	0.42
MII	0.03	0.01	0.006
Number of embryos transferred	−0.013	0.02	0.6
Day of transfer			
Day 3			0.35
Day 5	−0.03	0.03	
Pattern			
Pattern 1	–	–	
Pattern 2	−0.04	0.04	
Pattern 3	0.01	0.06	<0.001
Pattern 4	0.13	0.04	

*LBR, live birth rate; BMI, body mass index; MII, metaphase II oocytes*.

## Discussion

To our knowledge, this retrospective study is the first to investigate cumulative LBR using ESHRE's different subpopulations of PORs. We stratified patients into more homogeneous sub-categories and found statistically significant differences in several baseline characteristics such as female age, AFC and AMH levels. Fresh and cumulative LBR differed significantly between the four patterns and according to our results, pattern 4, representing a younger subpopulation, with a better clinical prognosis. In other terms, we provide evidence that clinical heterogeneity, which in turn translates into different outcome prognosis, still exists within BC PORs.

The role of age as an independent predictor of reproductive outcomes has been previously highlighted, given the increase in the percentage of aneuploidy rates with age, being 30% in women younger than 35 years and rising over 90% in women older than 42 years (21, 22). In our study, despite a higher number of oocytes yielded and a lower cycle cancelation in pattern 3 (the only subgroup with normal ORT), the best clinical outcomes were observed in pattern 4, underlining the importance of age.

Following the publication of the BC in 2011, very few studies have been conducted to investigate their validity (23). Our results are in contrast with previous reports finding similar fresh LBR in different subgroups of BC PORs (13, 24). In particular, La Marca et al. (13)ĭncluded 210 PORs in a retrospective analysis and showed similar LBR ranging from 5.5 to 7.4% in all groups defined as follows: group 1 (two cycles with <4 oocytes) vs. group 2 (age > 40 years + cycle with <4 oocytes), vs. group 3 (age > 40 + abnormal markers of ovarian reserve), vs. group 4 (cycle with <4 oocytes + abnormal markers of ovarian reserve) vs. group 5 (cycle with <4 oocytes + age > 40 + abnormal markers of ovarian reserve). Similar findings were reported by Busnelli et al. (24), in a retrospective analysis of 362 women allocated to five subgroups generated using BC, specifically: (i) anamnestic risk factors and one previous poor ovarian response; (ii) anamnestic risk factors and an abnormal ORT; (iii) an abnormal ORT and one previous poor ovarian response; (iv) anamnestic risk factors, an abnormal ORT and one previous poor ovarian response; (v) two episodes of poor ovarian response after maximal stimulation. The analysis showed LBR around 6% and no differences among the various subgroups.

Several reasons may explain the discrepancy between our results and those reported by Busnelli et al. and La Marca et al. First, our sample size of 706 PORs is considerably larger, limiting the probabilities of type II error. Second, the difference in age of the younger subgroup of the previously described studies compared to our own could also be an explanation (i.e., median age was 38 ± 3.9 years vs. 35.3 ± 3.5 in La Marca et al. and our study, respectively). Additionally, the differences in the threshold used for ovarian reserve biomarkers and the gonadotropin starting dose may also account for these divergent results.

In contrast with the aforementioned findings, our results are in line with those described by Bozdag et al., who demonstrated LBR of 2.3–8.7% per started IVF cycle in BC PORs, with “young proven” PORs having the most favorable reproductive outcomes (12). Nonetheless, our primary endpoint was cumulative LBR, which is a more relevant clinical outcome, while Bozdag et al. assessed only fresh LBR (12).

The BC have been criticized for several reasons including the lack of clarity in defining risk factors for poor ovarian response and the fact that they do not account for oocyte quality and other factors that can be associated with diminished ovarian reserve (11, 23, 25, 26). However, the major issue remains the persistence of heterogeneity among patients that very often present with different baseline characteristics and therefore diverse prognoses. In this context, in yet another attempt to overcome the limitations of the BC, a modified definition of impaired ovarian response has been proposed by the Poseidon Group (Patient-Oriented Strategies Encompassing Individualized Oocyte Number) (27). These novel criteria categorize women to more homogeneous subgroups according to age, ORTs and oocyte yield in previous OS cycles. However, currently, there are no data comparing the clinical relevance of the Bologna and the Poseidon criteria and further research is warranted.

The major strength of the current study relies on its large sample size and the choice of cumulative LBR as primary outcome. The inclusion of PORs according to BC is another positive appraisal point, given the recent reluctance of fertility experts in using the BC in studies regarding poor ovarian response (23). Furthermore, the exclusive use of an antagonist protocol to achieve pituitary suppression translates into increased homogeneity, which helps in drawing valid conclusions.

Nonetheless, the retrospective study design should be considered a limitation. Although a significant effort has been made to eliminate all known sources of systematic error through multivariable analysis allowing adjustment for relevant confounders, non-apparent sources of bias might still persist. In addition, given the difficulty to pool data relevant to other risk factors related to POR, we only analyzed 4 out of the possible 8 BC POR subpopulations (11). Finally, although the study design was fairly robust regarding the fresh cycles, frozen ET preparation was not consistent among the population. However, frozen cycle protocols with natural or artificial preparation have been shown to be equally effective (28–31).

In conclusion, establishing the BC represented the first real attempt of the medical community to reduce the vast heterogeneity underlying the definition of POR that greatly limited extrapolation of results from different studies and validation of conclusions. However, despite a proven applicability in subsequent research, the BC include patients with diverse baseline characteristics and therefore different clinical outcomes. The results of our study emphasize the discrepancy in clinical outcomes among the different subpopulations of PORs, with the younger subgroup having a better prognosis. Further modification of BC, perhaps by stratifying PORs in different subcategories that display more similar characteristics, may improve the shortcomings of the present definition.

## Data Availability Statement

The datasets generated for this study are available on request to the corresponding author.

## Ethics Statement

The studies involving human participants were reviewed and approved by Ethics Committee of Brussels University Hospital. Written informed consent for participation was not required for this study in accordance with the national legislation and the institutional requirements.

## Author Contributions

ARo, JE, and AK were responsible for data extraction and collection. ARo and PD were responsible for the statistical analysis. ARo, EB, and PD were responsible for writing the manuscript. SS-R, MV, ARa, SM, CB, PP, AV, and HT have all contributed in the interpretation and editing of the manuscript. PD was responsible for the concept and the final revision of the article.

### Conflict of Interest

The authors declare that the research was conducted in the absence of any commercial or financial relationships that could be construed as a potential conflict of interest.
