# Supplementary material for: Heterogeneity Among Poor Ovarian Responders According to Bologna Criteria Results in Diverging Cumulative Live Birth Rates
Source: Front Endocrinol (Lausanne). 2020 Apr 16;11:208. doi: 10.3389/fendo.2020.00208 (PMC7179754; doi:10.3389/fendo.2020.00208)
Supplement: Supplementary file 1 [file Data_Sheet_1.docx]

**Supplementary table 1. Reproductive outcomes (accordingly with AFC)**

|  | Pattern 1  age≥40 y  AFC<7  (n=275) | Pattern 2  age≥40 y  AFC<7  PS ≤3 COCs  (n=171) | Pattern 3  age≥40 y  PS ≤3 COCs  (n=49) | Pattern 4  age<40 y  AFC<7  PS≤3 COCs  (n=280) | *P* value |
| --- | --- | --- | --- | --- | --- |
| Biochemical pregnancy rate, n (%) | 51(19) | 28(16) | 12(24) | 54(19) | 0.62 |
| Clinical pregnancy rate, n (%) | 47(17) | 23(13) | 10(20) | 47(17) | 0.59 |
| Ongoing pregnancy rate, n (%) | 21(8) | 6(4) | 4(8) | 40(14) | 0.001 |
| LBR, n (%) | 21(8) | 6(4) | 4(8) | 39(14) | 0.001 |
| Cumulative LBR, n (%) | 24(9) | 6(4) | 6(12) | 50(18) | <0.001 |

LBR: live birth rate
