# Supplementary material for: Heterogeneity Among Poor Ovarian Responders According to Bologna Criteria Results in Diverging Cumulative Live Birth Rates
Source: Front Endocrinol (Lausanne). 2020 Apr 16;11:208. doi: 10.3389/fendo.2020.00208 (PMC7179754; doi:10.3389/fendo.2020.00208)
Supplement: Supplementary file 2 [file Data_Sheet_2.docx]

|  | Pattern 1  (n=293) | Pattern 2  (n=111) | Pattern 3  (n=31) | Pattern 4  (n=266) | *P* value |
| --- | --- | --- | --- | --- | --- |
| Biochemical pregnancy rate, n (%) | 54(18) | 22(19) | 4(13) | 49(18) | 0.877 |
| Clinical pregnancy rate, n (%) | 49(16) | 20(17) | 2(6) | 43(16) | 0.023 |
| Ongoing pregnancy rate, n (%) | 20(7) | 6(5) | 1(3) | 41(15) | <0.001 |
| LBR, n (%) | 20(7) | 6(5) | 1(3) | 40(15) | 0.001 |
| Cumulative LBR, n (%) | 23(8) | 6(5) | 2(6) | 49(18) | <0.001 |

**Supplementary table 2 Reproductive outcomes (one cycle per patient)**

LBR: live birth rate
